# Impairments in action–outcome learning in schizophrenia

**DOI:** 10.1038/s41398-018-0103-0

**Published:** 2018-03-03

**Authors:** Richard W. Morris, Chad Cyrzon, Melissa J. Green, Mike E. Le Pelley, Bernard W. Balleine

**Affiliations:** 1 School of Psychology, UNSW Sydney, Sydney, NSW Australia; 2 Brain & Mind Centre, University of Sydney, Sydney, NSW Australia; 3 ARC Centre of Excellence in Cognition and its Disorders, Macquarie University, Sydney, NSW Australia; 4 School of Psychiatry, UNSW Sydney, Sydney, NSW Australia; 5 Neuroscience Research Australia, Sydney, NSW Australia

## Abstract

Learning the causal relation between actions and their outcomes (AO learning) is critical for goal-directed behavior when actions are guided by desire for the outcome. This can be contrasted with habits that are acquired by reinforcement and primed by prevailing stimuli, in which causal learning plays no part. Recently, we demonstrated that goal-directed actions are impaired in schizophrenia; however, whether this deficit exists alongside impairments in habit or reinforcement learning is unknown. The present study distinguished deficits in causal learning from reinforcement learning in schizophrenia. We tested people with schizophrenia (SZ, *n* = 25) and healthy adults (HA, *n* = 25) in a vending machine task. Participants learned two action–outcome contingencies (e.g., push left to get a chocolate M&M, push right to get a cracker), and they also learned one contingency was degraded by delivery of noncontingent outcomes (e.g., free M&Ms), as well as changes in value by outcome devaluation. Both groups learned the best action to obtain rewards; however, SZ did not distinguish the more causal action when one AO contingency was degraded. Moreover, action selection in SZ was insensitive to changes in outcome value unless feedback was provided, and this was related to the deficit in AO learning. The failure to encode the causal relation between action and outcome in schizophrenia occurred without any apparent deficit in reinforcement learning. This implies that poor goal-directed behavior in schizophrenia cannot be explained by a more primary deficit in reward learning such as insensitivity to reward value or reward prediction errors.

## Introduction

The capacity to detect the causal effects of our actions is a critical prerequisite of goal-directed learning, allowing our actions to be regulated by their consequences^1–4^. When actions are goal-directed, then they are guided by desire for the outcome, as well as the belief that a particular action will cause that outcome. This excludes another class of adaptive behavior that is not mediated by anticipation of the goal but instead learned by a process of gradual reinforcement and primed by contextual stimuli or recent response history; that is, *habit learning* or (model-free) reinforcement learning^5–8^. Schizophrenia is associated with slow acquisition of adaptive behavior and inflexible responses^9–11^; however, little evidence exists to establish whether this is due to an impairment in goal-directed learning or habit learning^12^.

Recently, we reported a deficit specifically related to goal-directed learning in schizophrenia using an outcome devaluation test, a definitive criterion of goal-directed behavior^13^. Participants learned to select between two actions (pushing a vending machine to the left or to the right) leading to different snack food outcomes (sweet M&M chocolates or salty crackers). After training on these two *action*–*outcome* (AO) relationships, one of the outcomes was devalued (e.g., M&Ms were shown to be infested by cockroaches). The effect of this devaluation on participants’ behavior was then tested. This test revealed people with schizophrenia were relatively unaffected by devaluation; they tended to select actions on the basis of their original preferences before devaluation. One implication of these findings is that goal-directed learning is impaired in schizophrenia, whereas reinforcement learning remains relatively intact^14–18^. If schizophrenia is associated with a specific impairment in goal-directed learning, then procedures that distinguish the influence of causality from reward value should also selectively distinguish this deficit. One such procedure is contingency degradation.

When two AO contingencies are learned concurrently, participants can distinguish the effect of each action because each action produces a different outcome (i.e., left button produces M&Ms and right button produces crackers). Selective contingency degradation will occur when the base rate of one outcome, but not the other, is increased by delivering that outcome in the absence of any action (“noncontingent” outcomes). Importantly, the delivery of noncontingent outcomes will diminish the reward value of both actions equally since reward can now be obtained without making either action. Hence the effect of noncontingent outcomes on the overall rate at which rewards are received (the *reinforcement rate*) will apply equally to both actions. However, the noncontingent outcome will selectively degrade the *causal* relationship of only one action and not the other. This occurs because the noncontingent outcome is indistinguishable from the outcomes caused by one action but easily distinguishable from the outcomes caused by the other action. That is, earned M&Ms are easily distinguishable from crackers (earned or free) but hard to distinguish from noncontingent M&Ms. Thus any preference for the action with unique consequences (i.e., the non-degraded action) indicates a preference for causal actions rather than non-causal ones, a preference that cannot be ascribed to differences in reinforcement rate.

The aim of the present study was to establish whether participants with schizophrenia (SZ) could distinguish the causal consequences of their actions, as distinct from simply learning about the reward value of their actions (i.e., reinforcement learning). See Supplementary Figure 1 for an overview of the design. We initially trained participants with two AO contingencies whose reward value changed across blocks, to first confirm that SZ could learn and distinguish different reward contingencies (i.e., reward contingency learning). Any group differences apparent in reward contingency learning would be consistent with a reinforcement learning deficit in schizophrenia. We then selectively degraded the causal relationship of one AO contingency by delivering its outcome in the absence of any action, to assess causal learning (contingency degradation). Differences (within-subject) between degraded and non-degraded actions on this task indicate each participant’s sensitivity to the causal consequences of their actions, and we expected to reveal a selective impairment in causal learning in schizophrenia (i.e., smaller differences between degraded and non-degraded actions among SZ than healthy adults (HA), without any overall differences between groups). We then aimed to determine any relationship to goal-directed behavior by devaluing one of the outcomes (outcome devaluation) and testing whether changes in reward value could be integrated with action selection without feedback, before a final test with feedback. This study design allows further interrogation of these processes than other studies by establishing whether a selective deficit in causal learning, rather than reinforcement learning, exists in schizophrenia, as well as the extent to which this contributes to poor goal-directed behavior.

## Methods and materials

All participants provided written informed consent according to the approval requirements of the Human Research Ethics Committee of the University of Sydney (HREC #12812).

### Participants

Twenty-five HA and 25 people with schizophrenia or schizoaffective disorder (SZ) and no other Axis 1 disorder were included after meeting the inclusion criteria. Nine SZ participants had previously participated in an outcome devaluation test, as reported in Morris et al. (2015), and so were excluded from the outcome devaluation assessment reported here. The remaining 16 SZ and all HA participants were naive. SZ had a lifetime diagnosis of schizophrenia (*n* = 16) or schizoaffective disorder (*n* = 9) according to Diagnostic and Statistical Manual of Mental disorders, Fourth Revision criteria^19^. See Table 1 for demographic characteristics.

**Table 1:** Mean (SD) clinical and neuropsychological results

	Schizophrenia (*n* = 25)	Healthy (*n* = 25)	*t*-value (df = 48)	*p*-Value
Age, years	45 (8)	41 (15)	1.04	.30
Females	11	11		
Years of education	14 (2)	15 (2)	0.65	.52
WTAR IQ	104 (15)	111 (9)	1.65	.11
DASS-21 scores				
Depression	15 (9)	5 (7)	4.34	<.001
Anxiety	12 (9)	3 (6)	4.37	<.001
Stress	16 (10)	6 (5)	3.96	<.001
BIS/BAS scores				
BIS	22 (4)	20 (4)	1.64	.11
BAS-reward subscale	16 (3)	16 (2)	0.24	.81
BAS-drive subscale	11 (4)	10 (2)	0.95	.35
BAS-fun seeking subscale	10 (3)	10 (3)	0.18	.86
SAPS	24 (16)			
SANS	31 (11)			
Antipsychotics (CPZ mg/day)^a^	225 (115)			

*WTAR* Wechsler Test of Adult Reading, *DASS-21* Depression Anxiety Stress Scale 21 item version, *BIS/BAS* behavioral inhibition/approach system, *SAPS/SANS* Scale for the Assessment of Positive/Negative Symptoms, *CPZ* chlorpromazine equivalent dose

^a^Antipsychotic drug treatment: aripiprazole *n* = 3; clozapine *n* = 8; haloperidol *n* = 2; olanzapine *n* = 6; pallidperidone *n* = 1; quetiapine *n* = 1; risperidone *n* = 3;

### Reward Contingency Learning

The initial instrumental task was presented with the following instructions:

“Someone has told you that free snacks can be taken from the vending machine on the next screen. Use the button box to tilt the machine to the left or right, and try to find the best action to earn snacks. Please use only one finger to press buttons.”

During each 60-s block, the entire time was divided into brief (1-s) time bins that were unsignaled and therefore hidden to the participant so it appeared as a free-operant task. Left and right button presses were assigned different outcomes (e.g., left = M&Ms, right = crackers, counterbalanced between participants) and this relationship was held constant throughout the entire experiment for each participant. During the Reward Contingency Learning stage, left and right button presses produced their contingent outcomes at either a relatively high or a low probability per second: *p*(O|A)^high^ = 0.2 and *p*(O|A)^low^ = 0.05. These probabilities were held constant for the duration of each block but changed between blocks in an ABBABA order. That is, the left button press produced outcomes with a high probability in blocks 1, 4, and 6, and with a low probability in blocks 2, 3, and 5 (while the opposite order was applied to the right button press). By varying the better action (i.e., the button associated with the higher AO contingency) from block to block, we were able to determine whether participants could learn to adapt their responding based on prevailing reward contingencies, rather than a pre-existing preference for one outcome (or action) over another. Outcomes were indicated by the presentation of a visual stimulus depicting the relevant food for 1-s duration at the end of the 1-s time bin (rather than immediately after the winning button press). Actions made by the participant during the 1-s period that the outcome was displayed produced an animation on screen (i.e., the vending machine tilted) and were recorded for analysis but could not produce reinforcement. Participants were required to make all responses using a single finger of one hand (thus making it impossible to press both buttons simultaneously). Furthermore, only the most recent action in each second was considered for reinforcement so that both actions could not be rewarded in a single 1-s time bin. No rewards were delivered if a button had not been pressed [i.e., *p*(O|~A) = 0]. At the end of each 60-s block, participants rated how causal each action was with respect to its outcome, on separate 7-point Likert scales for each action, from 1 (not at all) to 7 (very causal). Participants completed six blocks of the Reward Contingency Learning stage.

### Contingency Degradation

The Contingency Degradation test began with the instructions:

“The vending machines on the next screen are malfunctioning and sometimes release one of the snacks at random. Use the button box to tilt the machine to the left or right and try to discover which action still causes snacks to drop. Please use only one finger to press buttons.”

In contrast to the previous stage, the left and right button presses were now reinforced with equal probabilities [*p*(O_i_|A_i_) = 0.2 and *p*(O_j_|A_j_) = 0.2]. One outcome was also provided at the same probability when no action had occurred for 1 s [i.e., *p*(O_i_|~A_i_, ~A_j_) = 0.2], and hence ∆*p* = 0 for the *degraded* AO relationship. The other snack was never delivered if no action had occurred [*p*(O_j_|~A_i_, ~A_j_) = 0], and hence ∆*p* = 0.2 for the contingent AO relationship. The identity of the free snack (O_i_ or O_j_) was varied from block-to-block in an ABBABA order. In this manner, we arranged to degrade the causal relationship for one AO in each block, while ensuring two important features: (1) there was no serendipitous contingency between an action and a free outcome, which would result in a higher reward contingency for the degraded action^20^, and (2) the earned outcome appeared at a varying interval up to 1 s after a successful action, which is sufficient to introduce ambiguity into the perceived AO contingency^21^. At the end of each 60-s block, causal ratings of each action were collected as described earlier. Six blocks of actions and ratings were collected in this manner during the Contingency Degradation stage.

### Outcome Devaluation

The Outcome Devaluation test occurred for a subset of naive SZ participants (*n* = 16) and a matching subset of healthy controls (*n* = 16). This test represented a replication of our earlier study^13^, and so we calculated the required sample size to observe the same group effect in an a priori fashion using G*Power^22^. The observed group difference between action and choices after outcome devaluation in Morris et al. had effect size Cohen’s *d* = 1.26, so with alpha = 0.05 and power = 0.90, the required group size for replication was *n* = 15. Each participant was verbally instructed that something had happened to one of the snacks and they watched a movie for 4 min depicting one of the snack foods (counterbalanced) infested with cockroaches. The outcome devaluation test began with the instructions:

“You can tilt the vending machine on the next screen for different snacks. The amount you earn will be recorded and you will eat what you have earned at the end of the experiment.”

On the next screen, the vending machine was presented and participants responded freely; however, no outcomes were presented for 30-s. After this non-reinforced test interval, outcomes appeared on screen as they were earned, with the same contingency for both AO relationships (∆*p* = 0.2). The reinforced test interval lasted for 2 min.

For details of pre- and post-test food ratings, as well as data analysis, see Supplemental information.

## Results

### Reward Contingency Learning

**Fig. 1:**
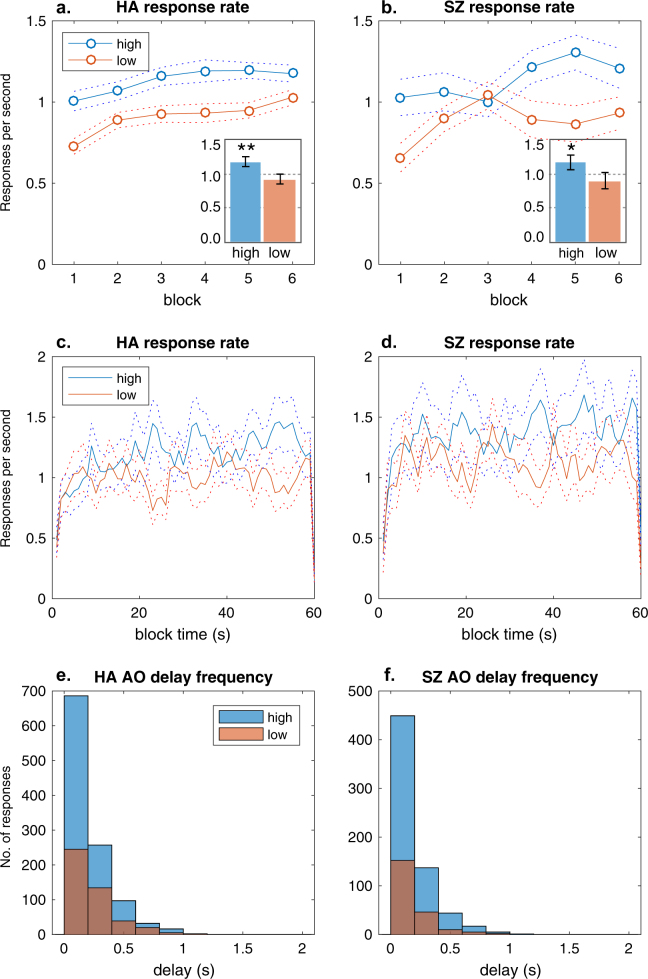
Contingency results. (**a**) Mean (SEM) rate of responding per second on the high-contingency action was higher than the low-contingency action across the six blocks in healthy adults (HA). Inset shows the mean (SEM) rate of responding per second over the test. **p < .01. **b** Mean (SEM) rate of responding per second on the high-contingency action was higher than the low-contingency action in people with schizophrenia (SZ). Inset shows the mean (SEM) rate of responding over the test. *p < .05. **c** Mean (SEM) rate of responding per second on the high and low-contingency action within blocks reflected a similar pattern in HA and **d** in SZ. **e** The delay between action and outcome was similarly distributed between 0 and 1 s for high and low-contingency actions in HA and **f** in SZ

**Fig. 2:**
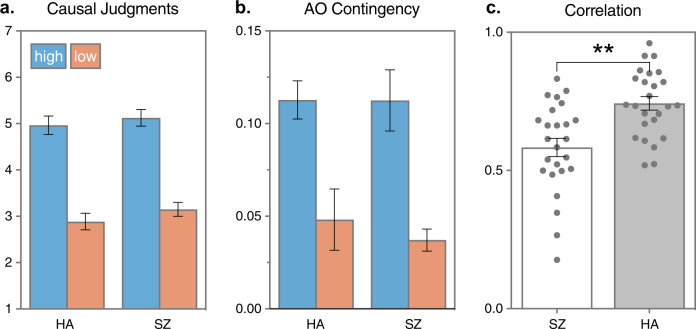
Reward contingency results. **a** Mean (SEM) causal ratings obtained after each block of the high-contingency action was higher than the low-contingency action in healthy adults (HA) and people with schizophrenia (SZ). **b** Mean (SEM) action–outcome contingencies (post hoc) experienced by each group confirmed that the high-contingency action was higher than the low-contingency action. **c** Mean (SEM) correlation (Pearson *r*) between causal judgments and experienced contingency was greater for HA than for SZ, **p < .01. Individual correlations shown as scatter dots

In each 1 min block, HA and SZ were able to learn the best action, selecting the high-contingency action more than the low-contingency action and rating it as more causal. Figure 1a, b show that response rates (per second) were higher for the high-contingency action than for the low-contingency action in each group across the six blocks, with little apparent difference between groups. Nor were there group differences apparent in the overall response rates calculated from the total number of presses over the test, shown in the insets of Fig. 1a, b. Figure 1c, d show that the pattern of responding within blocks, calculated for each hidden 1-s time bin, reveals little apparent difference between groups. A 2 × 2 repeated-measures (RM) multivariate analysis of variance (MANOVA) on the overall response rates, with group (HA vs SZ) and action (high vs low) included as factors, confirmed that the high-contingency action was preferred overall, main effect of action (*F*_1,48_ = 176.44, $\eta _p^2$ = 0.80, *p* < .001). Neither the main effect of group nor the interaction were significant (*F*s < 1, $\eta _p^2$ < 0.01, *p*s > .90). Figure 1e, f confirmed that the distributions of the AO delay frequencies experienced by each group were also similar. Figure 2a shows both groups rated the high-contingency action as more causal at the end of each block, with no apparent group differences. An analogous 2 × 2 RM MANOVA on ratings confirmed that the main effect of action was significant (*F*_1,48_ = 223.57, $\eta _p^2$ = 0.84, *p* < .001). Neither the main effect of group nor interaction for the causal judgments were significant. Figure 2b shows that the experienced contingencies for the programmed high- and low-contingency action were equally distinct for each group (main effect of action *F* = 91.375, $\eta _p^2$ = 0.05, *p* < .001), confirming we had sufficient experimental control in this stochastic free-operant task. These results provide no evidence that the SZ group were slower to learn new contingencies and are consistent with intact reinforcement learning and/or intact goal-directed learning in schizophrenia.

Because AO learning relies on awareness of the causal relationship between the action and the outcome, we determined whether the causal judgments of each participant varied with the experienced AO contingencies. Figure 2c shows the individual Pearson correlations (*r*) between causal judgments and AO contingency, as well as the mean of each group. While causal judgments and experienced contingency positively varied for each participant, the mean *r* among HA was significantly higher than among SZ (*t*_*48*_ = 3.48, *p* = .001, *d* = 0.15, 95% confidence interval (CI) [0.06, 0.23]). This indicates that, despite similar levels of performance, the causal judgments by SZ were not influenced by the AO contingency to the same extent as HA, suggesting that there may be a deficit in goal-directed learning (alongside intact reinforcement learning) in schizophrenia.

### Contingency Degradation

**Fig. 3:**
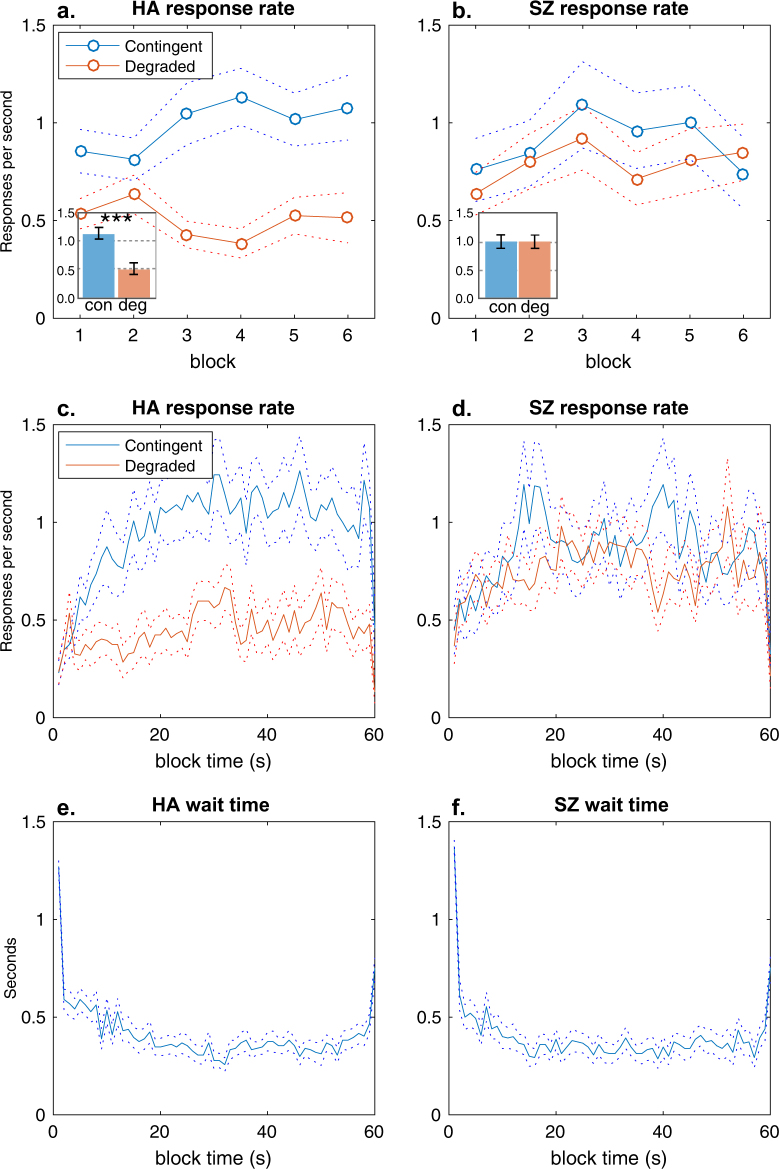
Degradation results. **a** Mean (SEM) rate of responding on the degraded action over the six blocks was less than the other (contingent) action in healthy adults (HA) but **b** not in people with schizophrenia (SZ). Inset shows the mean (SEM) rate of responding over the test. ***p < .001. **c** Mean (SEM) rate of responding within blocks reflected the same decrease on the degraded action in HA but **d** not in people with schizophrenia. **e** Mean (SEM) time (s) spent waiting during blocks for HA and **f** people with schizophrenia

In contrast to Reward Contingency Learning, SZ displayed a clear deficit in causal learning when one AO contingency was degraded by non-contingent (free) outcomes. Figure 3 shows that the mean response rates for the degraded action were lower than for the other (contingent) action among HA, indicating successful AO learning as healthy people preferred the more causal action (Fig. 3a, 3a inset, c). However, this clear preference did not appear among SZ in either the response rates across blocks (Fig. 3b), overall (Fig. 3b inset) or within block (Fig. 3d). The 2 × 2 RM MANOVA, with group (HA vs SZ) and action (Deg vs Con) included as factors, confirmed that the preference for the non-degraded (Contingent) action varied with group interaction (*F*_1,48_ = 9.77, $\eta _p^2$ = 0.17, *p* = .003). Follow-up *t*-tests confirmed that the group by action interaction was due to a significantly higher response rate on the contingent action than the degraded action in HA (*t*_24_ = 3.85, *d* = 0.83, *p* = .0008). This pattern did not interact with block (*F*_5,235_ = 1.13, *p* = .34), and there was no significant main effect of group (*F*_1,48_ = 1.09, $\eta _p^2$ < 0.02, *p*s > .30). These data indicate a selective deficit in causal action selection in schizophrenia. Figure 3e, f show this deficit was not due to a failure to inhibit responding as both groups spent similar amounts of time not taking either action (“waiting”) within sessions. Furthermore, there was no significant group difference in the total number of outcomes (non-contingent or contingent) received by each group (*t*_48_ = 1.45, *p* = .16). Supplementary Fig. S2A shows that there were no group difference in either the total or the distribution of free outcomes received by each group: all participants received free outcomes (lowest total was 5), and SZ (*M* = 4.07, SEM = 2.18) received slightly more on average than HA (*M* = 3.56, SEM = 2.18), but this was not significant (*t*_48_ = 0.97, *p* = .34). There was no significant correlation between the rate of responding on the contingent action and total reward received for either groups (HA *r* = –.05, *p* = .99; SZ *r* = + .07, *p* = .98), confirming that the preference for the action cannot be ascribed to differences in reinforcement. Finally, Supplementary Figure S2B and S2C show the distribution of the AO delay differed between the degraded and contingent AO, but there were no significant group differences in the distributions of delays experienced by each group (lowest *p* = .36; two-sample Kolmogorov–Smirnov test).

**Fig. 4:**
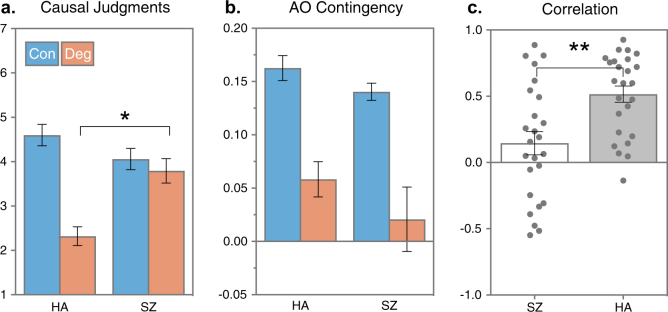
Degradation results. **a** Mean (SEM) causal ratings obtained after each block of the degraded and contingent action indicated people with schizophrenia (SZ) judged the degraded action as more causal than healthy adults (HA). *p < .05. **b** Mean (SEM) action–outcome contingencies (post hoc ∆*P*) experienced by each group confirmed both groups received similar AO contingencies during the degradation test. **c** Mean (SEM) correlation (Pearson *r*) between causal judgments and experienced contingency (∆*P*) was greater for HA than for SZ, **p < .01

Figure 4a shows that the causal judgments of the degraded action were clearly reduced by the non-contingent outcomes among HA but not among SZ (group by action interaction *F*_1,48_ = 14.61, $\eta _p^2$ = 0.23, *p* < .001). There was no interaction with block (*F*_5,235_ = 0.33, *p* = .89). The follow-up *t*-test confirmed that causal judgments of the degraded action were higher among SZ than HA (*t*_48_ = 3.82, *p* < .001, *d* = 1.06, 95% CI [0.51, 1.65]). The limits of the effect size CI indicate that the true group difference is likely to be moderate to large (in standardized units), demonstrating that sufficient power existed to reliably detect the effect size observed (*d* = 1.06). Furthermore, Fig. 4b shows these group differences were not due to serendipitous differences in the AO contingency (∆*p*) experienced by each group. The experienced ∆*p* on the contingent and degraded action confirmed ∆*p* was higher for the contingent action among HA (*M*_D_ = 0.11, SEM_D_ = 0.02) as well as people with schizophrenia (*M*_D_ = 0.12, SEM_D_ = 0.03), and there were no significant group differences (*t*_*48*_ = 0.41, *p* = .68, *d* = 0.12, 95% CI [−0.69, 0.45]). Finally, as in the reward contingency test, Fig. 4c shows the individual Pearson *r* correlations between causal judgments and AO contingency, as well as the mean of each group. Not surprisingly, there was a clear and significant group difference indicating the causal judgments of SZ were not as influenced by the experienced AO contingency as HA (*t*_48_ = 3.27, *p* = .002, *d* = 0.35, 95% CI [+0.14, +0.57]). In sum, the contingency degradation test revealed that SZ did not detect the degraded causal relationship when free outcomes occurred and instead tended to judge (and select) their actions as if both AO relationships were equally causal.

### Outcome Devaluation

**Fig. 5:**
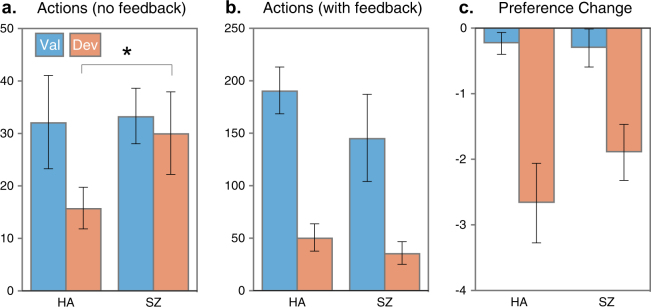
Devaluation results. **a** Mean (SEM) number of actions during the outcome devaluation test without feedback, and SZ showed a higher preference for the devalued action than HA *p < .05, and **b** Mean (SEM) number of actions during the outcome devaluation test with feedback. **c** Change in preference scores (Post−Pre) for the non-devalued (Val) outcome and the devalued outcome among the subset of HA (*n* = 16) and SZ (*n* = 16)

Figure 5a, b show participants’ actions during the initial period of the non-reinforced test interval (i.e., in which no feedback was provided, see Morris et al.^13^, and the subsequent reinforced test interval in which feedback was provided (a novel extension of our previous study). The non-reinforced test replicated the deficit in goal-directed actions in schizophrenia that we have reported previously^13^; i.e., without feedback, HA were more likely to make the action associated with the valued outcome rather than the devalued outcome, by contrast SZ were equally likely to make both actions regardless of the value of the associated outcome. The proportional preference for the valued action among HA (*M* = 0.67, SEM = 0.04) was significantly higher than among SZ (*M* = 0.50, SEM = 0.05; *t*_33_ = 2.17, *p* = .02, Cohen’s *d* = 0.74, 95% CI [0.16, 1.31]). The proportional preference of 0.50 found for SZ represents no effect of devaluation. Although the effect size observed in the current study was somewhat smaller than what we found previously, this nevertheless constitutes a successful replication (note the effect size CI for the group difference includes the original effect size of 1.26 reported in Morris et al.^13^. Once feedback was provided in the reinforced test session, a significant preference for the action associated with the valued outcome emerged in SZ (*M* = 0.67, SEM = 0.06) along with HA (*M* = 0.79, SEM = 0.04), and it did not differ significantly between groups (*t*_33_ = 1.34, *p* = .19, Cohen’s *d* = 0.46, 95% CI [−0.23, 1.15]).

### Pre- vs post-test food ratings

Figure 5c shows that devaluation reduced the post-test food preference ratings in both groups: the mean (±SEM) change in the rating for the devalued food was −1.89 (±0.42) and −2.67 (±0.61) for HA and SZ, respectively. The mean (±SEM) change in rating for the other (non-devalued) food was significantly less, −0.30 (±0.17) and −0.23 (±0.28) for HA and SZ, respectively, indicating that devaluation selectively reduced preference ratings for the infested snack for both groups, (*t*s = 3.75 and 3.72, *p*s < .01).

### Relationship between causal learning and goal-directed actions

We determined the strength of the correlation between causal learning and goal-directed actions. The difference score between choices from the degradation and (nonreinforced) devaluation tests, for causal learning and goal-directed actions, respectively, were correlated among participants who had performed both. The correlation between difference scores for degradation and devaluation was Pearson’s *r* = .66 (df = 23, *p* < .001) for HA and *r* = .73 (df = 14, *p* < .001) for SZ, indicating that differences in causal learning explain almost half the variance in goal-directed actions in each group.

### Symptom, medication and mood effects in SZ

Table 2 shows the results of a correlation analysis between AO learning and symptom, medication, and mood states in SZ. There was a significant correlation between avolition scores (Scale for the Assessment of Negative Symptoms subscale) and valued over devalued actions during the devaluation test (Table 2, *r* = –.49, *p* = .02), consistent with the inverse relationship between goal-directed actions and negative symptoms we have previously reported^13^. We also found an inverse relationship between anxiety and causal actions during degradation among SZ (Table 2, *r* = –.56, *p* = .008), such that AO learning improved with lower levels of anxiety. Because we have previously observed an inverse relationship between functional outcomes in schizophrenia and goal-directed choices^13^, we also tested for negative correlations with World Health Organization Disability Assessment Schedule (v2.0) scores within the SZ group. A moderate association existed between causal judgments and overall disability score, *r* = –0.40 (one-tailed *p* = .03), indicating that disability increased as AO learning declined in schizophrenia.

**Table 2:** Pearson correlations with causal learning and goal-directed choice

	Causal judgments	Causal actions	Goal-dir. choices	Reinf. choices
SANS subscales				
Affect	0.25	0.14	−0.09	−0.43
Alogia	0.33	−0.04	−0.17	−0.35
Avolition	−0.11	−0.14	−0.35	−0.49*
Anhedonia	−0.10	−0.15	−0.30	−0.22
Attention	0.09	0.03	−0.18	−0.05
SAPS subscales				
Hallucinations	−0.08	0.03	0.15	0.06
Delusions	−0.15	−0.12	0.14	0.11
Bizarre behavior	−0.30	−0.28	−0.40	−0.42
Positive thought disorder	−0.38	−0.37	−0.17	−0.01
Antipsychotics (CPZ mg/day)	0.07	0.17	−0.02	−0.36
DASS-21				
Depression	−0.13	−0.02	0.18	−0.01
Anxiety	−0.41	−0.56**	0.05	−0.26
Stress	−0.35	−0.23	0.03	−0.47
WHODAS	−0.40^	−0.36	−0.35	−0.29

Causal judgments: Difference scores (∆) between ratings for non-degraded and degraded actions; Causal actions: Difference scores (∆) between response rates for non-degraded and degraded actions; Goal-dir. choices: Difference scores (∆) between non-devalued and devalued actions during the nonreinforced test period; Reinf. choices: Difference scores (∆) between non-devalued and devalued actions during the reinforced test period

*SAPS/SANS* Scale for the Assessment of Positive/Negative Symptoms, *CPZ* chlorpromazine equivalent dose, *DASS-21* Depression, Anxiety and Stress Scale (21 item version), *WHODAS* World Health Organization Disability Assessment Schedule (v2.0)

**p < .01, *p < .05, ^*p* < .05 one-tailed

## Discussion

The present study demonstrates a specific deficit in learning the causal relationship between actions and outcomes in schizophrenia without any observable deficit in reinforcement learning. In the initial reward contingency test, the SZ group successfully learned which of two actions resulted in a higher probability of reinforcement within each block (Fig. 1c, d). Furthermore, both groups adapted to the changes in the reward contingencies which took place over blocks, on the basis of feedback alone (Fig. 1a, b), in a manner consistent with conventional theories of reinforcement learning^7,8^. The causal judgments obtained at the end of each block also indicated that both groups were equally aware of the best action, since both groups rated the high-contingency action as more causal (Fig. 2a). However group differences were revealed in the correlation between causal judgments and the experienced contingency of each participant (Fig. 2c). That is, while both groups learned the best action and seemed to be equally aware of the best action, causal judgments were more closely related to the experienced contingency among HA than among SZ. AO learning is based on an acquired belief about the causal efficacy of our actions on the basis of experience^23^, and so the concordance between causal judgments and the experienced contingency in HA is consistent with intact AO learning. However, the lower concordance among actions and outcomes in SZ suggests that, despite similar levels of performance and awareness, instrumental performance may be mediated by a somewhat different mechanism in schizophrenia.

The subsequent Contingency Degradation test provided the critical evidence that instrumental performance in schizophrenia is not the result of AO learning. This test required participants to learn the best action when the probability of reinforcement was the same for both actions but the causal relationship between one action and outcome pair was degraded by the delivery of noncontingent outcomes. We observed a clear bias in HA toward the more causal, non-degraded action (Fig. 3a, c), which suggests action selection was guided by causal learning. Importantly, there were no post hoc differences between actions in the probability of reward nor the correlation between the action taken and total reward, which confirms that we successfully equated the contiguity with reward while varying causal efficacy of the two actions. Among SZ, we did not observe a similar preference for the more causal action (Fig. 3b, d); this was not due to differences in the distribution of free rewards, the distribution of AO delays, or differences in time spent waiting for free rewards (Figure S2). Furthermore, SZ did not judge the degraded action as less causal but instead rated it more causal than HA, consistent with a failure of causal learning alongside intact reinforcement learning (insert comment about response rate = causal judgment) (Fig. 4a).

We argue that these results represent novel evidence that schizophrenia is associated with a specific deficit in encoding causal actions, which is not confounded by differences in reinforcement. The reward contingency stage and the contingency degradation stage share many of the same cognitive requirements, thus the fact that patients successfully learnt the reward contingencies in the initial stage rules out a number of non-specific explanations of the causal learning deficit. For instance, the adaptive behavior displayed by patients during the reward contingency task demonstrated that they have the working memory capacity to hold in mind the best action, as well as the behavioral flexibility to pursue it within each block. We believe this establishes better evidence of their ability to perform the non-specific features of the causal learning task than additional neuropsychological tests of working memory or executive function; however, we do not rule out the likelihood that such executive deficits may exist in our sample. Other latent differences between our groups, such as socioeconomic background and premorbid Intelligent Quotient, might also be relevant to the observed deficit. However, an analysis of covariance including Wechsler Test of Adult Reading scores and education levels as a covariate (reported in the Supplementary information) failed to explain the differences in performance during contingency degradation. Thus working memory capacity or other problems of executive function that may be present but not specific to causal learning seem unlikely to provide an account of the deficit we report here.

Previous studies have reported aberrant instrumental learning in schizophrenia using tasks that do not distinguish the contribution of the causal relationship from reinforcement or, for that matter, from deficits in reinforcement learning per se^12^. For example, schizophrenia has been associated with aberrant perseveration in reversal learning tests as well as poor performance in probabilistic instrumental learning tests^15,18,24–26^. On the basis of such results, researchers have argued that schizophrenia is characterized by impairment in some aspect of reinforcement learning or reward prediction, typically related to the rapid learning of changes in response contingencies (e.g., ref. ^18^). We did not observe a slower rate of acquisition among patients (e.g., Figs. 1c, d and 3c, d) nor did group differences significantly interact with block effects. Nevertheless, we argue that an impairment in encoding the specific AO associations, alongside intact stimulus–response (habit) learning, is consistent with an early deficit in learning without asymptotic differences in performance. However, this pattern would only appear in tasks which confound the two processes since AO learning is typically dominant in the early stages of performance rather than the later stages^1,27,28^. In tasks which separate the influence of AO learning from reinforcement/habit learning, then the deficit will be task-specific, as we observed here.

The impairment in learning the causal efficacy of actions in schizophrenia is likely to contribute to the deficit in goal-directed action that we reported previously^13^ and replicated here (Fig. 5a). Goal-directed actions can be distinguished from habitual responses by the role the outcome plays in action selection. When action selection is goal-directed, the value of the outcome determines choice. On the other hand, when responding is habitual, it is mediated by stimulus–response associations for which the outcome value plays no part. For this reason, the outcome devaluation test represents a direct test of goal-directed actions, as it measures the influence of outcome value on choice; however, it also requires the integration of recent changes in outcome value with prior causal learning. We report here that almost half the variance in performance in an outcome devaluation test was associated with causal learning, which implies that much of the goal-directed deficit in schizophrenia may be due to a problem with causal learning. The remaining unexplained variance may indicate that an independent problem with integrating value with action selection also exists in schizophrenia. Collectively, the emerging evidence from this study as well as our previous work suggests that the goal-directed deficit in schizophrenia is due to more than one impairment: that is, a deficit in integrating changes in outcome value with action selection, as well as a potentially independent deficit in causal learning that also results in a failure to integrate value with action selection. Successful remediation of poor goal-directed behavior in schizophrenia will depend on correct identification of the primary impairment in each case.

Other researchers have described the impairments that characterize goal-directed learning in schizophrenia slightly differently. For example, it has been argued that people with schizophrenia can learn simple stimulus–response associations to guide action selection, but a deficit in representing *predicted reward value* impairs goal-directed choices^9,29,30^. This is somewhat different from our proposal that the deficit exists in encoding the specific *consequences of actions*. On the basis of our initial reward contingency test, we would argue that patients are able to learn the response that predicted greater reward. These findings (for review, see ref. ^15^) indicate that people with schizophrenia can learn the expected value of their responses (i.e., action values). We distinguish our account on the basis of how those values are represented: that is, we would argue that the value of an outcome and its sensory features (sight, taste, smell, etc) can be distinct and separately represented. Accordingly, the impairment in schizophrenia appears to reflect a failure to encode the sensory features of an action’s consequences (i.e., the outcome). As a result, the outcome value is acquired by the action rather than encoded with the outcome, and this results in inflexible performance when the value of the outcome changes. A similar distinction is made in model-based vs model-free learning, but we emphasize that the deficit occurs in the sensory representation of the outcome rather than representing the (expected) value.

A specific deficit in learning the consequences of one’s actions has implications for understanding the neuropathology of schizophrenia. Substantial evidence from contingency degradation tests in humans and rodents show that the medial prefrontal cortex (prelimbic cortex) is critical to detect whether or not an action is causally related to an outcome^31–34^. Disruption to either the dopaminergic innervation to this region or its excitatory outputs to the dorsal striatum renders actions insensitive to their degraded causal status^28,34^. Imaging studies in humans have also confirmed that a similar corticostriatal network exists between the medial prefrontal cortex (mPFC) and caudate to track the causal efficacy of actions^1,3,35^. We might therefore expect that the antidopaminergic effect of medication would impact causal learning in the present study; however, we found no relationship between antipsychotic drug dose (CPZ scores) and causal learning among patients. However, our previous work implicates pathology in the inputs to the caudate in schizophrenia during goal-directed choices^13^, although it did not reveal the cortical source of those inputs. The same mPFC circuit has been implicated in learning about control over aversive events, where damage to this circuit can exacerbate learned helplessness and abolish the resilience to stress normally seen after escapable shock training^36,37^. Dysfunction in this circuit would have wide-reaching consequences for dealing with stress as well as goal-directed learning, since patients are less likely to experience control over aversive events in their day-to-day living. The self-reported anxiety ratings and disability scores of patients appear to support this view, since anxiety and disability increased with severity of the causal learning deficit. However, given the number of correlations we tested without correction for multiple comparisons, we must interpret the somewhat post hoc nature of these associations with caution until they are replicated in studies that are more optimally designed for investigating individual differences.

In sum, we present evidence that a specific deficit related to encoding the causal consequences of one’s actions exists in schizophrenia, which contributes to a more general deficit in goal-directed learning. People (and animals) must be able to understand the consequences of their actions in order to select actions on the basis of those consequences. This is distinct from reinforcement learning by which people and animals acquire a response by repetition, and which perhaps explains patients' high response rates on the degraded action and their subsequent overestimated causal judgments. The impairment in learning the causal efficacy of actions is likely to contribute to the deficit in goal-directed action that we reported previously in schizophrenia^13^ and replicated here (Fig. 5a). Collectively, the emerging evidence from our work and others suggests that the goal-directed deficit in schizophrenia is due to pathology in converging inputs to the caudate, perhaps from the mPFC. Dysfunctional connectivity in this corticostriatal path results in a failure to learn the causal effects of actions, thus setting the stage for a perceived lack of control and ultimately helplessness and avolition in schizophrenia.

## Electronic supplementary material

Supplementary Figures

Supplemental information
