# Supplementary material for: Impairments in action–outcome learning in schizophrenia
Source: Transl Psychiatry. 2018 Mar 3;8:54. doi: 10.1038/s41398-018-0103-0 (PMC5834614; doi:10.1038/s41398-018-0103-0)
Supplement: Supplementary file 1 — Supplementary Figures [file 41398_2018_103_MOESM1_ESM.pdf]

Supplementary Figure 1

a. Contingency Learning

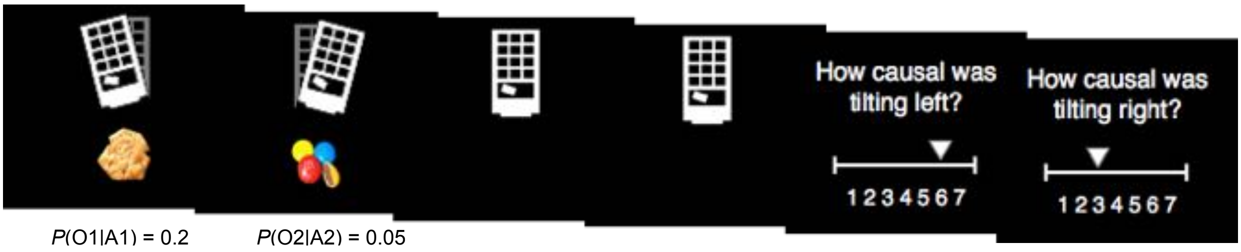

b. Contingency degradation

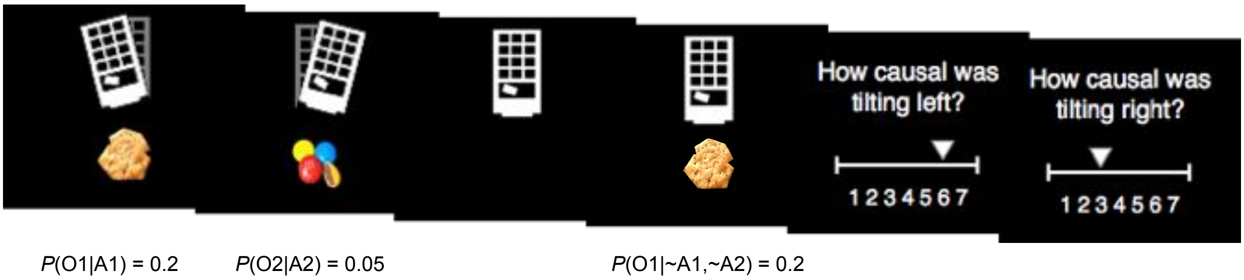

c. Outcome devaluation

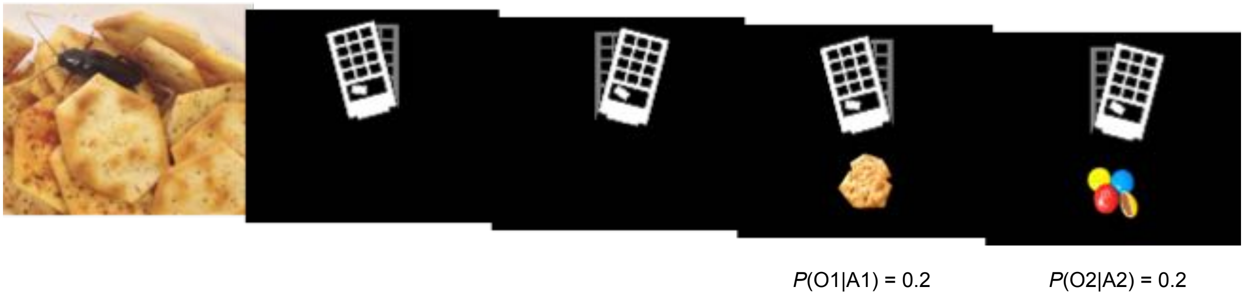

Figure S1. Stimulus screens in **a)** reward contingency learning **b)** contingency degradation and **c)** outcome devaluation tests. Tests were designed to exclude any discriminative cues that might indicate the best action, in order to minimize the influence of Pavlovian learning and emphasize action-outcome learning

Supplementary figure 2. Degradation Results

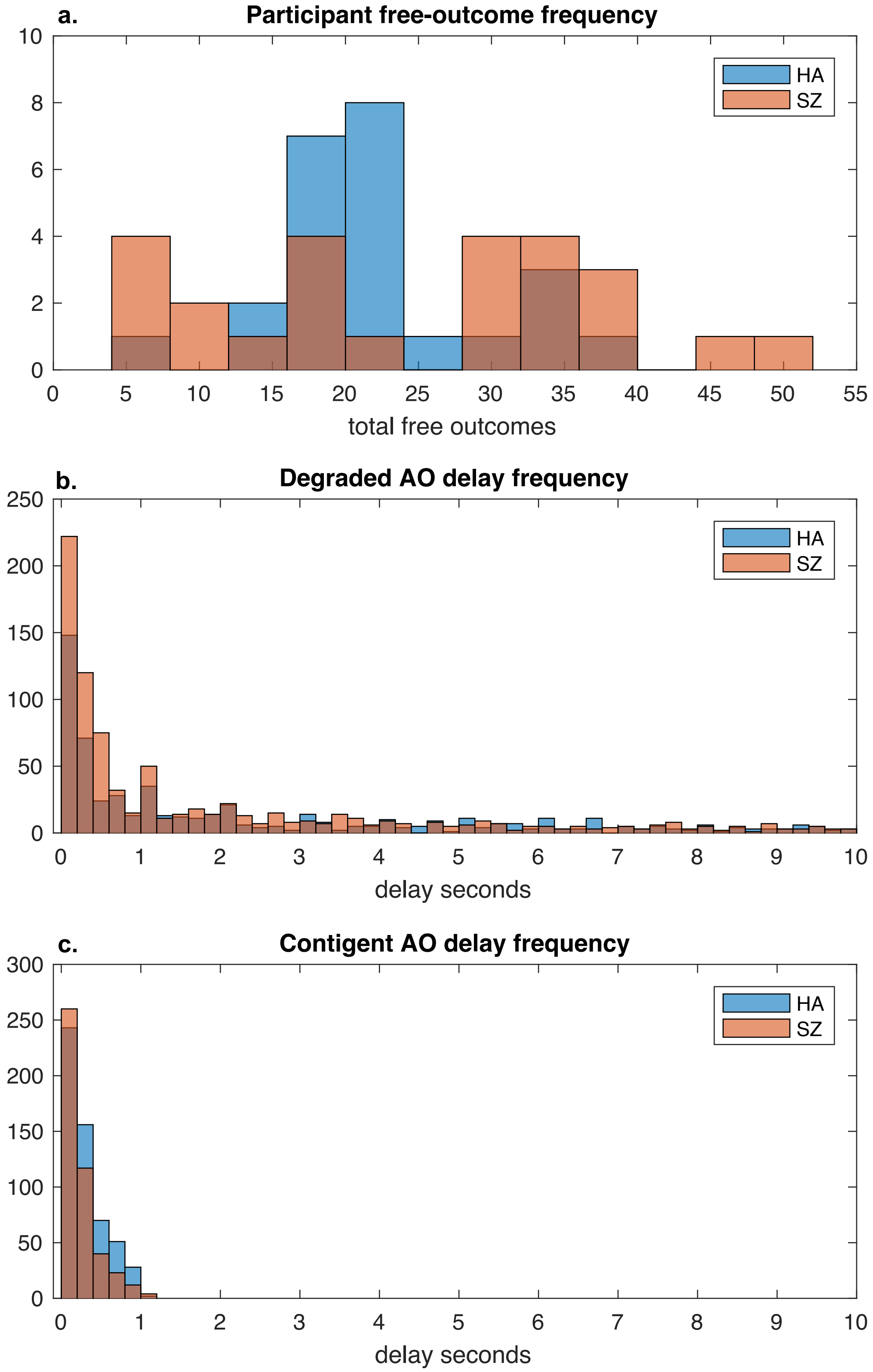

Figure S2. Degradation Results. **a)** Histograms of the total non-contingent (free) outcomes received by each participant were similar for both groups. **b)** Frequency distributions of the degraded action-outcome delays experienced by each group were similar for both groups, and **c)** the distributions of the contingent action-outcome delays experienced by each group were also similar

Supplementary figure 3. Rating Results by Block

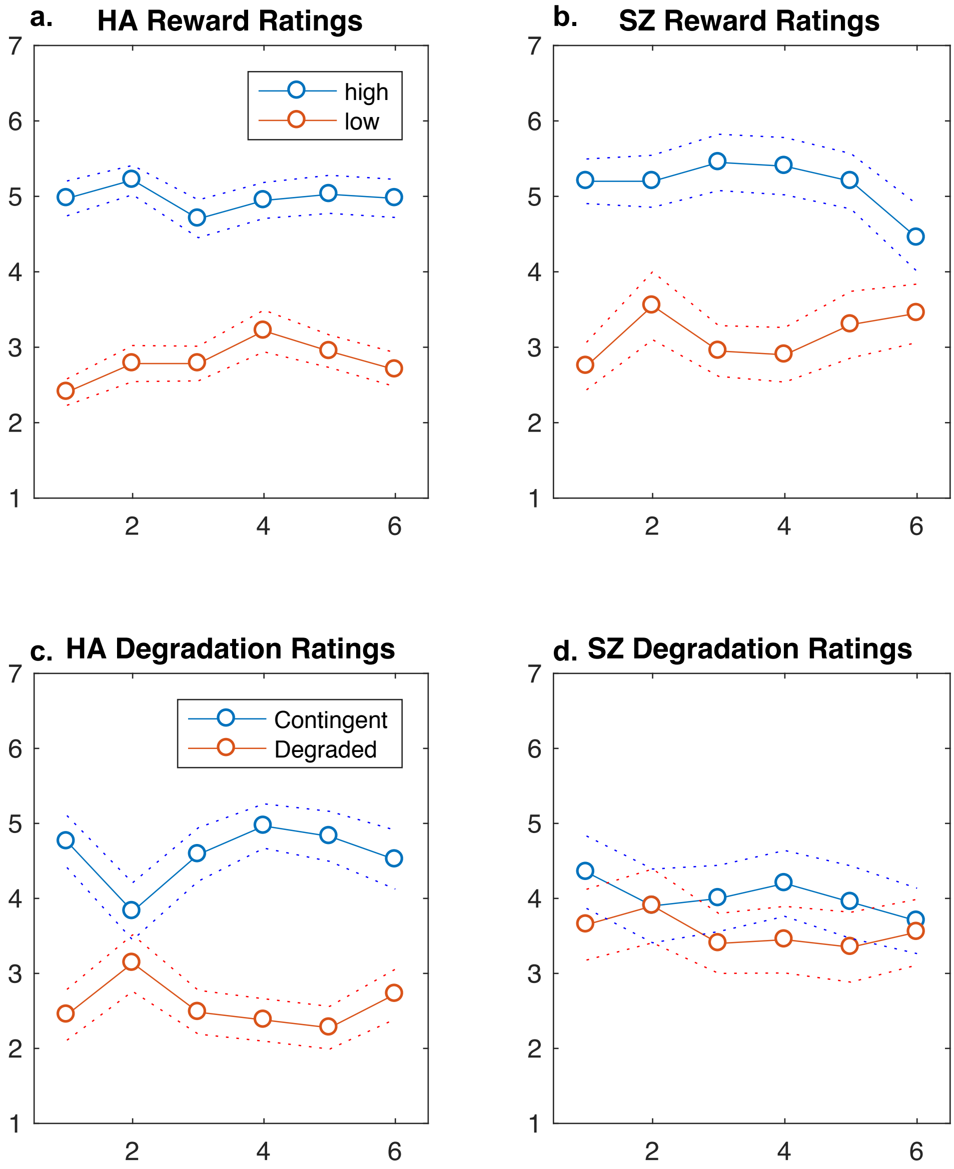

Figure S3. Mean ratings from the judgments at the end of each of the six blocks from **a)** Reward Contingency test in healthy adults **b)** and Reward Contingency test in people with schizophrenia **c)** Contingency Degradation test in healthy adults and **d)** Contingency Degradation test in people with schizophrenia
