# Supplementary material for: Impairments in action–outcome learning in schizophrenia
Source: Transl Psychiatry. 2018 Mar 3;8:54. doi: 10.1038/s41398-018-0103-0 (PMC5834614; doi:10.1038/s41398-018-0103-0)
Supplement: Supplementary file 2 — Supplemental information [file 41398_2018_103_MOESM2_ESM.docx]

# Supplemental Information

Methods and Materials

All participants provided written informed consent according to the approval requirements of the Human Research Ethics Committee of Sydney University (HREC #12812).

**Participants**. For all participants, inclusion criteria were age between 21 and 55 years, no food allergies and no substance abuse during the past 6 months, and a high self-reported preference for at least two of the snack foods we used as rewards. In addition, participants were excluded for failure to understand or remember the task instructions. Participants were recruited through the Australian Schizophrenia Research Bank (1). Healthy adults had no personal or family history of psychosis. Premorbid IQ was assessed using the Wechsler Test of Adult Reading (WTAR) (2). Positive and negative symptoms were rated using the Scales for Assessment of Positive and Negative Symptoms (SAPS/SANS) (3). Self-report questionnaires collected demographic information including gender, age, medication, drug use and World Health Organisation Disability Assessment Schedule (WHODAS) (4), and the Depression, Anxiety and Stress Scale (DASS-21) (5).

**Stimuli**. In each experiment the two highest rated snack foods were used as outcomes, selected from among a choice of BBQ flavoured crackers (Arnott’s®, Australia), M&M™ chocolate candy (Mars, Australia) and Tiny Teddy™ cookies (Arnott’s®, Australia). During the task an image of the snack food was displayed on-screen but snacks were provided only at the end of all testing; no snack foods were available during testing. Participants made actions (tilt left or right) using a custom-made, two-button response box connected to a laptop computer. Stimulus presentation and response recording was controlled by PsychoPy (v1.74.03) (6, 7) running on a MacBook™ computer (Apple, CA). A diagram of the test screen is shown in Supplementary Figure 1.

**Pre- & post-test food ratings.** Before training began, the three different food rewards were presented and participants rated how much they wanted each snack item right now on a 7-point Likert scale (“not at all” to “extremely”). At the end of testing, post-test preference ratings were collected for each snack using the 7-point Likert scale.

**Data analysis**. To determine group differences in AO learning, response rates and causal judgments were analyzed in a 2 x 2 repeated measures MANOVA with group (HA vs SZ) and action (e.g., Degraded vs Contingent) as between- and within-subject factors respectively. The analysis was implemented in R (v3.3.2). A general deficit in reinforcement learning in schizophrenia would be indicated by a significant group main effect reflecting lower performance due to slower learning in SZ relative to HA. In contrast, a selective deficit in causal learning among people with schizophrenia would be indicated by a significant group by action interaction, reflecting a smaller difference between contingent and degraded actions (or judgments) in SZ compared to HA. Post-hoc t-tests were used to confirm the source of the interaction. Partial eta squared $\eta_{p}^{2}$or Cohen’s *d* is reported as in-sample effect size estimates for each group difference. For each planned group t-test we calculated 95% confidence intervals in standardized units using a bootstrapping procedure (Cumming & Finch, 2005), to provide out-of-sample effect size estimates (12, 13).

Pearson’s correlation coefficient (*r*) was used to assess the relationship between AO learning and causal judgments, symptoms, medication and mood effects. To calculate these correlations, AO learning was indicated by the difference score between actions (or judgments) (e.g., contingent action – degraded action; high action – low action). That is, AO learning in the reward contingency stage was calculated by ∆response =  high reward responses – low reward responses, and ∆rating = high reward rating – low reward rating. Similarly AO learning in the contingency degradation stage was calculated as ∆action = non-degraded action – degraded action and ∆rating = non-degraded rating – degraded rating. The Pearson *r* was then calculated between ∆response and ∆rating, and between ∆action and ∆rating for each person across the six blocks in each stage.

We did not include block as a factor in the ANOVAs described above so as to avoid the complication of interpreting a 3-way interaction. However we tested whether the effect of block interacted with any of the significant group differences in a 2 x 6 RM MANOVA, with group (HA vs SZ) and block (1 to 6) as between- and within-subject factors respectively. Instead of including action, these analyses were performed on the difference scores between actions: High – Low or Con – Deg actions. In this manner we could still test whether block or group affected the differences in actions without interpreting a 3-way interaction.

Results

**Pre-Test Food Ratings**. Participants were screened for a high self-reported preference for the snack foods and so pre-test food preference ratings provided after sampling each food were similarly high for both groups: The mean (±SEM) affective rating for BBQ crackers among HA and SZ was 5.5 (±0.60) and 5.33 (±0.32), respectively; for M&Ms was 6.2 (±0.8) and 5.73 (±0.26), respectively; and for Tiny Teddy cookies was 4.5 (±0.2) and 4.93 (±0.39), respectively. Two-sample t-tests confirmed no significant group differences in pre-test ratings (lowest *p* = .32).

**Symptom, Medication and Mood Effects in SZ**. We examined whether the deficit in AO learning, indicated by the difference score between the degraded and contingent ratings or actions, was related to symptoms, medication or mood effects in SZ (see Table 2). We found no significant correlations between symptom scores (individual subscale scores in the SAPS and SANS) and AO learning among SZ during the degradation test. There were no significant relationships between AO learning and stress or depression as measured by the DASS, or antipsychotic drug dose (CPZ equivalent).

We also tested whether the group differences in actions and judgments survived in an ANCOVA model, after accounting for the variance due to either WTAR or education level. Difference scores were calculated between actions (Con – Deg actions) for ratings and responses, and we included the covariate (either WTAR or education) before the group factor in each of four ordered ANCOVA models (Type 1). In these models, the covariate took priority and so the model provided a test of whether the effect of group survived in the remaining variance. In each case, the effect of group was significant, *F*_1,47_ = 8.33, 11.51, 8.57 and 10.14 (*p*s = .005, .001, .005, and .002), for the two models of responses with WTAR and education as covariates and for the two models of ratings with WTAR and education as covariates, respectively. The effect of the covariate was not significant in any of these models, highest *F*_1,47_ = 3.06, *p* = .086 for WTAR in the model of responses.

Bibliography

1. Loughland C, Draganic D, McCabe K, Richards J, Nasir A, Allen J, et al. Australian Schizophrenia Research Bank: a database of comprehensive clinical, endophenotypic and genetic data for aetiological studies of schizophrenia. The Australian and New Zealand journal of psychiatry. 2010;44(11):1029-35.

2. Wechsler D. Wechsler Test of Adult Reading. San Antonio, Texas: The Psychological Corporation; 2001.

3. Andreasen NC. Negative symptoms in schizophrenia: Definition and reliability. Archives of General Psychiatry. 1982;39(7):784-8.

4. Gold LH. DSM-5 and the assessment of functioning: the World Health Organization Disability Assessment Schedule 2.0 (WHODAS 2.0). The journal of the American Academy of Psychiatry and the Law. 2014;42(2):173-81.

5. Lovibond SH, Lovibond PF. Manual for the Depression Anxiety Stress Scales. 2nd ed. Sydney: Psychology Foundation; 1995.

6. Peirce JW. PsychoPy—Psychophysics software in Python. Journal of Neuroscience Methods. 2007;162(1–2):8-13.

7. Peirce JW. Generating stimuli for neuroscience using PsychoPy. Frontiers in Neuroinformatics. 2009;2.

8. Dickinson A, Mulatero CW. Reinforcer Specificity of the Suppression of Instrumental Performance on a Non-Contingent Schedule. Behav Process. 1989;19(1-3):167-80.

9. Shanks DR, Dickinson A. Instrumental judgment and performance under variations in action-outcome contingency and contiguity. Memory & cognition. 1991;19(4):353-60.

10. Morris RW, Quail S, Griffiths KR, Green MJ, Balleine BW. Corticostriatal control of goal-directed action is impaired in schizophrenia. Biological Psychiatry. 2015;77(2):187-95.

11. Faul F, Erdfelder E, Buchner A, Lang AG. Statistical power analyses using G*Power 3.1: tests for correlation and regression analyses. Behavior research methods. 2009;41(4):1149-60.

12. Cumming G. Understanding the New Statistics: Effect sizes, Confidence Intervals, and Meta-Analysis. New York, NY: Routledge; 2012.

13. Lakens D. Calculating and reporting effect sizes to facilitate cumulative science: a practical primer for t-tests and ANOVAs. Frontiers in psychology. 2013;4:863.
